# IFN-*γ* Licensing Does Not Enhance the Reduced Immunomodulatory Potential and Migratory Ability of Differentiation-Induced Porcine Bone Marrow-Derived Mesenchymal Stem Cells in an *In Vitro* Xenogeneic Application

**DOI:** 10.1155/2021/4604856

**Published:** 2021-09-04

**Authors:** Hyeon-Jeong Lee, Hwan-Deuk Kim, Chan-Hee Jo, Eun-Yeong Bok, Saet-Byul Kim, Sung-Lim Lee, Min Jang, Seul-Gi Bae, Sung-Ho Yun, Seung-Joon Kim, Gyu-Jin Rho, Won-Jae Lee

**Affiliations:** ^1^College of Veterinary Medicine, Gyeongsang National University, Jinju 52828, Republic of Korea; ^2^College of Veterinary Medicine, Kyungpook National University, Daegu 41566, Republic of Korea; ^3^Department of Veterinary Research, Daegu Metropolitan City Institute of Health & Environment, Daegu 42183, Republic of Korea

## Abstract

IFN-*γ* licensing to mesenchymal stem cells (MSCs) is applied to enhance the therapeutic potential of MSCs. However, although the features of MSCs are affected by several stimuli, little information is available on changes to the therapeutic potential of IFN-*γ*-licensed differentiated MSCs during xenogeneic applications. Therefore, the present study is aimed at clarifying the effects of adipogenic/osteogenic differentiation and IFN-*γ* licensing on the *in vitro* immunomodulatory and migratory properties of porcine bone marrow-derived MSCs in xenogeneic applications using human peripheral blood mononuclear cells (PBMCs). IFN-*γ* licensing in differentiated MSCs lowered lineage-specific gene expression but did not affect MSC-specific cell surface molecules. Although indoleamine 2,3 deoxygenase (IDO) activity and expression were increased after IFN-*γ* licensing in undifferentiated MSCs, they were reduced after differentiation. IFN-*γ* licensing to differentiated MSCs elevated the reduced IDO expression in differentiated MSCs; however, the increase was not sufficient to reach to the level achieved by undifferentiated MSCs. During a mixed lymphocyte reaction with quantification of TNF-*α* concentration, proliferation and activation of xenogeneic PBMCs were suppressed by undifferentiated MSCs but inhibited to a lesser extent by differentiated MSCs. IFN-*γ* licensing increasingly suppressed proliferation of PBMCs in undifferentiated MSCs but it was incapable of elevating the reduced immunosuppressive ability of differentiated MSCs. Migratory ability through a scratch assay and gene expression study was reduced in differentiated MSCs than their undifferentiated counterparts; IFN-*γ* licensing was unable to enhance the reduced migratory ability in differentiated MSCs. Similar results were found in a Transwell system with differentiated MSCs in the upper chamber toward xenogeneic PBMCs in the lower chamber, despite IFN-*γ* licensing increased the migratory ability of undifferentiated MSCs. Overall, IFN-*γ* licensing did not enhance the reduced immunomodulatory and migratory properties of differentiated MSCs in a xenogeneic application. This study provides a better understanding of the ways in which MSC therapy can be applied.

## 1. Introduction

Mesenchymal stem cells (MSCs) are found in various adult tissues and organs including the bone marrow (BMMSCs), adipose tissue (ATMSCs), umbilical cord (UCMSCs), and dental pulp and are capable of self-renewal, differentiation to multilineages, migration into injured/damaged tissues, and immunomodulation toward immune cells via cell-to-cell contact or secretion of soluble factors [1–4]. Consequently, MSCs have been extensively investigated with the aim of utilizing their properties as a source of cell-based therapy, i.e., for tissue regeneration and replacement of the damaged tissue via their differentiation or immunomodulation capabilities, which can control immune responses in relation to cells of both innate and adaptive immunity [4–6]. Notably, MSCs have been experimentally applied in multiple disorders associated with orthopedic injury, myocardial defects, spinal/neuronal injuries, liver failure, and inflammatory mediated diseases including autoimmune diseases and graft-versus-host disease (GVHD) [5, 7, 8].

The immunomodulatory properties of MSCs are not innate features but are acquired in response to stimulation by proinflammatory cytokines such as interferon *γ* (IFN-*γ*), tumor necrosis factor *α* (TNF-*α*), and/or interleukin 1*β* (IL-1*β*); this process is known as “licensing” [6, 8]. Licensed MSCs mediate immunomodulation directly by cell-to-cell contact or indirectly by secreting chemokines such as C-C chemokine receptor (CCR)/ligand (CCL) and C-X-C chemokine receptor (CXCR)/ligand (CXCL), adhesion molecules like vascular cell adhesion molecule (VCAM) and intercellular adhesion molecule (ICAM), and immunosuppressive molecules including IL-10, indoleamine 2,3 deoxygenase (IDO), nitric oxide (NO), prostaglandin E2 (PGE2), tumor necrosis factor-stimulated gene (TSG), and transforming growth factor *β* (TGF-*β*) [7, 8]. In addition, MSCs induce the formation of regulatory T cells (Treg) that inhibit lymphocyte proliferation. Furthermore, MSCs trigger changes to the cytokine secretion profile and immune cell proliferation/activation in the innate immune system, including in dendritic cells (DCs), natural killer cells, and macrophages, as well as the adaptive immune system, such as in CD4+ T helper cells, CD8+ cytotoxic T cells, and B cells by suppressing the secretion of proinflammatory cytokines and increasing levels of anti-inflammatory cytokines [1, 4, 9]. With the aim of mimicking the inflammatory region or understanding the therapeutic mechanism of immunomodulation property-enhanced MSCs, several studies have utilized proinflammatory cytokine-licensed MSCs [4, 6, 10].

Because MSCs retain a property of less immunogenicity, or none at all, due to their low expression of major histocompatibility complexes (MHC-I and MHC-II) and T cell costimulatory molecules (CD40, CD80, and CD86), they can be administered by the allogeneic or xenogeneic application of cell transplantation [7, 11, 12]. In addition, safety, efficacy, and survival in the host after the application of MSCs have been validated during long-term follow-up studies in numerous clinical trials [1, 4]. Although many studies of MSC applications have used allogeneic transplantation to reveal their therapeutic mechanism in certain diseases or defects, xenotransplanted MSCs have also presented positive and ameliorating effects in systemic lupus erythematosus, collagen-induced arthritis, and cardiovascular disease [1, 7, 13]. In particular, the xenogeneic application of MSCs can be adopted as an unlimited cell source when cell numbers or donors are lacking during clinical trials or when cryopreserved MSCs are not requested [13].

Despite many studies indicated the therapeutic potential of MSCs, in some clinical trials, MSCs have shown little or no therapeutic efficacy [14]. Therefore, identifying the therapeutic applications of MSCs under various experimental conditions is still required [11]. The potential of MSCs is likely to be affected by internal and external stimuli, such as serial subcultures, stimulation by proinflammatory cytokines (IFN-*γ* and TNF-*α*), and differentiation induction; notably, differentiation induction in MSCs results in downregulation of apoptosis-related genes, shortening of telomere length with weakening of telomerase activity, changes to MSCs-specific cell surface molecules, and attenuation of immunomodulatory potential [2, 3, 15–18]. In addition, MSCs applied to the inflammatory sites are unavoidable to be exposed with proinflammatory cytokines. However, little information is available on changes to the immunomodulatory and migratory properties of differentiated MSCs with stimulation of proinflammatory cytokines in xenogeneic application models, despite both differentiation and proinflammatory cytokines are known to alter features of MSCs. Therefore, the aim of the present study was to assess changes in the immunomodulatory and migratory properties of differentiated porcine BMMSCs and to evaluate the effect of IFN-*γ* licensing on the differentiated MSCs, under a xenogeneic application model with human peripheral blood mononuclear cells (PBMCs).

## 2. Materials and Methods

### 2.1. Ethics Statement

Animal-related experimental procedures were approved by the Institutional Animal Care Use Committee at Kyungpook National University (approval number: 2021-0098).

### 2.2. Chemicals and Culture Media

Unless stated otherwise, all chemicals and culture media were purchased from Sigma-Aldrich Chemical Inc. (St. Louis, MO, USA) or Thermo Fisher Scientific (Waltham, MA, USA).

### 2.3. Cell Isolation of Porcine BMMSCs

To establish BMMSCs, the bone marrow extracts of the both femurs from approximately 6-month-old female domestic farm pigs who were not litter-mates (three­way crossbred by Landrace × Yorkshire × Duroc; body weight: approximately 120 kg; *n* = 4) were aspirated using bone marrow aspiration needles (Jamshidi, Becton, Dickinson and Company (BD), Franklin Lakes, NJ, USA) at the local abattoir after slaughter. Subsequently, extracts were immediately transferred to heparinized tubes (BD). Samples from healthy individuals were only collected with a veterinarian examination. Porcine BMMSCs (*n* = 4) were isolated from the mononuclear cell fractions of the bone marrow extract using centrifugation via the Ficoll (Ficoll Paque PLUS, GE Healthcare, Uppsala, Sweden) gradient method at 400 g and 4°C for 30 min, in accordance with previously published method [7, 19]. The cell fractions were then harvested and plated onto culture dishes with culture media consisting of advanced Dulbecco's modified Eagle medium containing 10% fetal bovine serum (FBS), 1% GlutaMax, 10 ng/mL basic fibroblast growth factor, and 1% penicillin–streptomycin. Once the adherent cells on the culture dishes were observed, the supernatant was discarded, and the culture media was replaced with fresh media. BMMSCs were cultured at 38.5°C in a humidified incubator with 5% CO_2_ and subcultured until passage 3 when ~90% cell confluence was reached.

### 2.4. Preparation of Experimental Groups

Undifferentiated BMMSCs at passage 4 were cultured with or without 50 ng/mL human IFN-*γ* (R&D systems, Minneapolis, MN, USA) supplementation during the 48 h prior to assessment; human IFN-*γ* was used to mimic a xenogeneic application of porcine MSCs to the human patients with inflammatory diseases. The concentration and licensing duration of IFN-*γ* followed methods in previous studies [6, 20]. In differentiation-induced groups, adipogenic or osteogenic differentiation of BMMSCs at passage 4 was induced in Dulbecco's modified Eagle's medium (DMEM) supplemented with 10% FBS, 100 mM indomethacin, 10 mM insulin, and 1 mM dexamethasone or DMEM supplemented with 10% FBS, 200 mM ascorbic acid, 10 mM *β*-glycerophosphate, and 0.1 mM dexamethasone, respectively [7, 19]. Under both differentiation induction conditions, the cells were cultured at 38.5°C in a humidified incubator with 5% CO_2_ for 2 weeks. Both differentiated BMMSCs were cultured with or without 50 ng/mL human IFN-*γ* (R&D systems) supplementation during the 48 h prior to completing differentiation inductions [3, 6, 20]. Adipogenic differentiation was identified by staining intracellular lipid vacuoles using 0.5% Oil red O solution. Osteogenic differentiation was confirmed through the accumulation of mineral deposits with staining by 5% sliver nitrate solution (Von Kossa staining) and 0.5% alizarin red solution. In addition, the expression of lineage-specific genes was evaluated in adipogenesis and osteogenesis using quantitative polymerase chain reaction (qPCR) with the relevant primers for adipogenesis (PPAR-*γ* and FABP-4) and osteogenesis (ON and OPN) (Table 1). Comprehensively, the porcine cells from six groups consisting of undifferentiated BMMSCs without IFN-*γ* supplementation (BMMSCs-IFN-*γ*) or with IFN-*γ* supplementation (BMMSCs+IFN-*γ*), differentiated BMMSCs toward adipocytes without IFN-*γ* supplementation (ADI-IFN-*γ*) or with IFN-*γ* supplementation (ADI+IFN-*γ*), and differentiated BMMSCs toward osteoblasts without IFN-*γ* supplementation (OST-IFN-*γ*) or with IFN-*γ* supplementation (OST+IFN-*γ*) were comparatively analyzed for expressions of MSC-specific surface molecules, alterations of the immunomodulatory potential, and changes in the migratory ability, under a xenogeneic application model with human PBMCs.

### 2.5. Assessment of MSC-Specific Surface Molecule Expression

The cells from six groups were analyzed for positive expression of MSC-specific markers (CD44, CD90, and CD105) and negative expression of a hematopoietic marker (CD45) using flow cytometry (BD FACS Calibur, BD), as previously described [7, 19]. The cells were harvested, filtered through 40 *μ*m meshes, fixed with 4% paraformaldehyde at 4°C overnight, and then blocked with 1% bovine serum albumin. The 1 : 200-diluted fluorescein isothiocyanate (FITC)-conjugated antibodies (BD) related to anti-CD44, anti-CD45, anti-CD90, anti-CD105, and an isotype control were labeled directly at room temperature (RT) for 1 h. Thereafter, 1 × 10^4^ FITC-labeled cells were counted with a flow cytometry.

### 2.6. Assessment of Gene Expression Study via qPCR

The qPCR-related procedures were conducted in accordance with those in previous reports [7, 19]. Total RNA was extracted from the six groups using a QIA shredder column and RNeasy Mini Kit (Qiagen, Hilden, Germany), including the RNase-free DNase treatment step for 15 min to remove residual genomic DNA. The concentration with purity of total RNA samples was quantified using an UV-Vis spectrophotometer (Nabi, MicroDigital Co., Ltd., Korea) via the A260/A280 ratio; only pure total RNA samples within a 2 ± 0.2 ratio were selected. First-strand cDNA was synthesized using 1 *μ*g total RNA, 4 units Omniscript Reverse Transcriptase (Qiagen), 10 units RNase inhibitor, and 1 mM oligo dT primer at 60°C for 1 h using a thermal cycler (Qiagen). The qPCR was conducted using a Rotor Gene Q qPCR machine (Qiagen) with Rotor-Gene 2× SYBR Green Mix (Qiagen), including 0.1 *μ*g cDNA per reaction and 0.5 mM forward and reverse primers (shown in Table 1). The qPCR program designed to obtain the cycle threshold (Ct) values was as follows: predenaturation at 95°C for 10 min, 40 PCR cycles at 95°C for 10 s, 60°C for 6 s, and 72°C for 4 s, melting curve from 60°C to 95°C at 1°C/s, and cooling at 40°C for 30 s. All transcriptional levels of target genes in Table 1 were normalized against a TBP, which is known as the most stable reference gene in porcine MSCs [19].

### 2.7. IDO Activity Measurements

Because kynurenine is known as the product in IDO-dependent catabolism of tryptophan, the IDO activity was assessed using the level of kynurenine derived from MSCs, as previously described [10, 20]. The culture media from six groups were centrifuged at 750 g for 5 min. The supernatant (100 *μ*L) was then harvested and mixed with 30% trichloroacetic acid (50 *μ*L), before being incubated at 50°C for 30 min. Thereafter, this solution was diluted with an equal volume of Ehrlich reagent (100 mg p-dimethylbenzaldehyde in 5 mL glacial acetic acid) and incubated at RT for 10 min. After the mixture was transferred to a 96-well plate, the absorbance at 492 nm was determined using a microplate reader (Molecular Devices, San Jose, CA, USA). The concentration of kynurenine was quantified using a serially diluted L-kynurenine standard curve.

### 2.8. Mixed Lymphocyte Reaction (MLR) with Xenogeneic PBMCs

To determine the immunosuppressive effect of undifferentiated and differentiated MSCs with INF-*γ* licensing in the xenogeneic application, MLR with human PBMCs (American Type Culture Collection, Manassas, VA, USA) was conducted in accordance with previously published methods [5, 12, 21]. In total, 1 × 10^5^ cells for each of the six groups were prepared as the effector, and these were treated with 10 *μ*g/mL of mitomycin C for 1 h at 37°C just prior to coculture with xenogeneic PBMCs. Human PBMCs were labeled with 5 *μ*M 5,6-carboxyfluorescein succinimidyl ester and suspended with the MLR culture media consisting of RPMI 1640 complete medium supplemented with 10% FBS, 1% penicillin–streptomycin, and 5 *μ*g/mL phytohemagglutinin (PHA), a mitogen for T cell proliferation. Overall, 1 × 10^6^ PHA-activated PBMCs were cocultured with MSCs from the six groups at an MSC : PBMC ratio of 1 : 10 in an MLR culture media at 37°C in a humidified incubator with 5% CO_2_ for 5 days. As a control, PHA-activated PBMCs seeded alone were cultured and counted as 100%. The PBMCs were then collected and levels of PBMC proliferation were assessed by flow cytometry (BD). All values were calculated as a percentage of the control.

### 2.9. Enzyme-Linked Immunosorbent Assay (ELISA) to Detection of TNF-*α*

Because TNF-*α* is known to be secreted from activated T cells [9], we assessed the level of TNF-*α* in the supernatant post-MLR assay with Quantikine® ELISA kits (R&D Systems) following manufacturer's protocol. In brief, standards and each supernatant post-MLR were incubated in wells precoated with the human primary antibody. The antigen-antibody complex was detected using human TNF-*α* conjugated with horseradish peroxidase. Thereafter, conjugates were quantified by a colorimetric reaction with a 3,3′,5,5′-tetramethylbenzidine substrate. The reaction intensities were read at 450 nm using a microplate reader (Molecular Devices) and calculated by interpolation from the standard curve.

### 2.10. Scratch Assay

The migration ability of cells was assessed using a scratch assay in accordance with previously published method [6]. Upon confirming confluence of cells for each of the six groups on a 6-well plate, the cell layer was scratched with 200 *μ*L sterile micropipette tips. After removing debris cells from the culture media, the adherent cells were incubated with FBS-free culture media for another 48 h at 37°C in a humidified incubator with 5% CO_2_. Images of scratched gaps were captured with an inverted microscope (Nikon, Japan), and the cell migration area was measured using Image J software (National Institutes of Health (NIH), USA). The closed area postscratch was normalized to the value obtained from BMMSCs-IFN-*γ* at 48 h.

### 2.11. Transwell System

Migratory ability in a Transwell system was evaluated using Matrigel coated 24-well Transwell plate with an 8.0 *μ*m polyester membrane (Corning Inc., Corning, NY, USA) as previously described [22, 23]. In total, 1 × 10^5^ cells for each of the six groups were prepared and seeded on the upper inserts with RPMI 1640 complete medium, whereas 1 × 10^5^ human PBMCs were added to the lower chambers with the same media composition during MLR culture. After incubation for 1 day at 37°C in a humidified incubator with 5% CO_2_, nonmigrated cells were removed from the upper side of the membrane with a cotton swab. Thereafter, the cells in the membranes were fixed with 100% methanol and stained with 0.2% crystal violet in 2% ethanol for 5 min at RT. Images of stained membranes were captured with an inverted microscope (Nikon) and measured using Image J software (NIH).

### 2.12. Statistical Analysis

All experiments were repeated in triplicate. Statistical significance was analyzed using one-way ANOVA via SPSS 12.0 (SPSS Inc. Chicago, IL, USA) followed by Games-Howell post hoc analysis. All data are presented as means ± standard error (SEM). *p* values of < 0.01 were considered statistically significant.

## 3. Results

### 3.1. Characterization of Porcine BMMSCs

**Figure 1:**
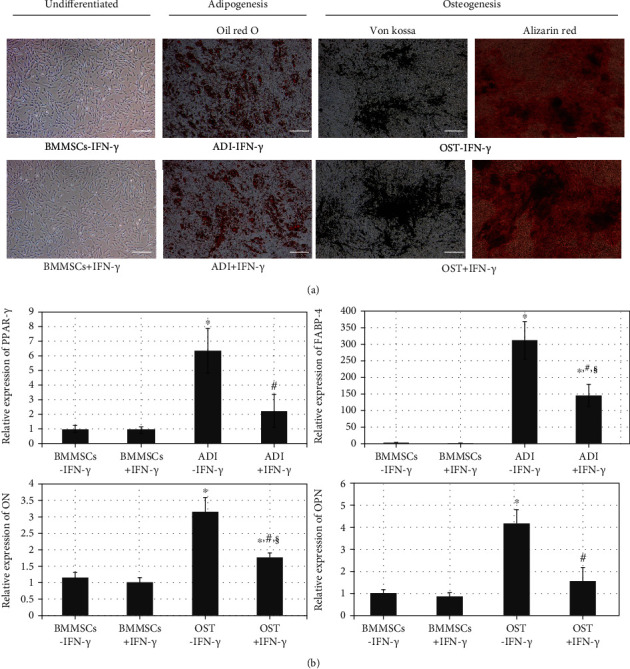
Characterization of porcine BMMSCs with or without IFN-*γ* licensing. (a) Morphology of undifferentiated BMMSCs with or without IFN-*γ* licensing (bright fields) and cytochemical observation of differentiated BMMSCs toward adipocytes (Oil red O) and osteoblasts (Von kossa and alizarin red) with or without IFN-*γ* licensing. Magnification: ×100. Scale bar: 100 *μ*m. (b) The expression of lineage-specific genes after differentiation toward adipocytes (PPAR-*γ* and FABP-4) and osteoblasts (ON and OPN). Data represent the means ± SEM. *X* and *Y* axes show types of cells and the relative expression of each gene, respectively. ^∗^*p* < 0.01 indicated significant difference from BMMSCs-IFN-*γ*. ^#^*p* < 0.01 indicated significant difference between each differentiated MSCs and their IFN-*γ*-licensed cells (ADI-IFN-*γ* vs. ADI+IFN-*γ* or OST-IFN-*γ* vs. OST+IFN-*γ*). ^§^*p* < 0.01 indicated significant difference between BMMSCs+IFN-*γ* and IFN-*γ*-licensed differentiated cells (BMMSCs+IFN-*γ* vs. ADI+IFN-*γ* or BMMSCs+IFN-*γ* vs. OST+IFN-*γ*). Abbreviations: BMMSCs: porcine bone marrow-derived mesenchymal stem cells; IFN-*γ*: interferon *γ*; BMMSCs-IFN-*γ* or BMMSCs+IFN-*γ*: undifferentiated BMMSCs without or with 50 ng/mL human IFN-*γ* supplementation during 48 h, respectively; ADI-IFN-*γ* or ADI+IFN-*γ*: differentiated BMMSCs toward adipocytes without or with 50 ng/mL human IFN-*γ* supplementation during 48 h, respectively; OST-IFN-*γ* or OST+IFN-*γ*: differentiated BMMSCs toward osteoblasts without or with 50 ng/mL human IFN-*γ* supplementation during 48 h, respectively; PPAR-*γ*: peroxisome proliferator-activated receptor gamma; FABP-4: fatty acid-binding protein 4; ON: osteonectin; OPN: osteopontin.

A plastic adherent cell population with a spindle shape and fibroblastic morphology was observed in the isolated porcine BMMSCs (BMMSCs-IFN-*γ*) and remained after IFN-*γ* licensing (BMMSCs+IFN-*γ*). In cytochemical observations, differentiated MSCs toward adipocytes or osteoblasts positively exhibited the accumulation of lipid droplets in Oil red O staining (ADI-IFN-*γ*) or mineral deposition (OST-IFN-*γ*) under Von Kossa and alizarin red staining, respectively; similar results were observed in IFN-*γ*-licensed differentiated MSCs (ADI+IFN-*γ* and OST+IFN-*γ*) (Figure 1(a)). In addition, lineage-specific gene expression after differentiation toward adipocytes (PPAR-*γ* and FABP-4) and osteoblasts (ON and OPN) was significantly (^∗^*p* < 0.01) upregulated compared with undifferentiated MSCs (BMMSCs-IFN-*γ* vs. ADI-IFN-*γ* or BMMSCs-IFN-*γ* vs. OST-IFN-*γ*); similar results were significantly (^§^*p* < 0.01) found in FABP-4 and ON expressions of IFN-*γ*-licensed groups (BMMSCs+IFN-*γ* vs. ADI+IFN-*γ* or BMMSCs+IFN-*γ* vs. OST+IFN-*γ*). However, IFN-*γ* licensing affected lineage-specific gene expression; it resulted in a significant (^#^*p* < 0.01) decrease in comparison with unlicensed differentiated MSCs (ADI-IFN-*γ* vs. ADI+IFN-*γ* or OST-IFN-*γ* vs. OST+IFN-*γ*) (Figure 1(b)).

### 3.2. Assessment of MSC-Specific Surface Molecule Expression

**Figure 2:**
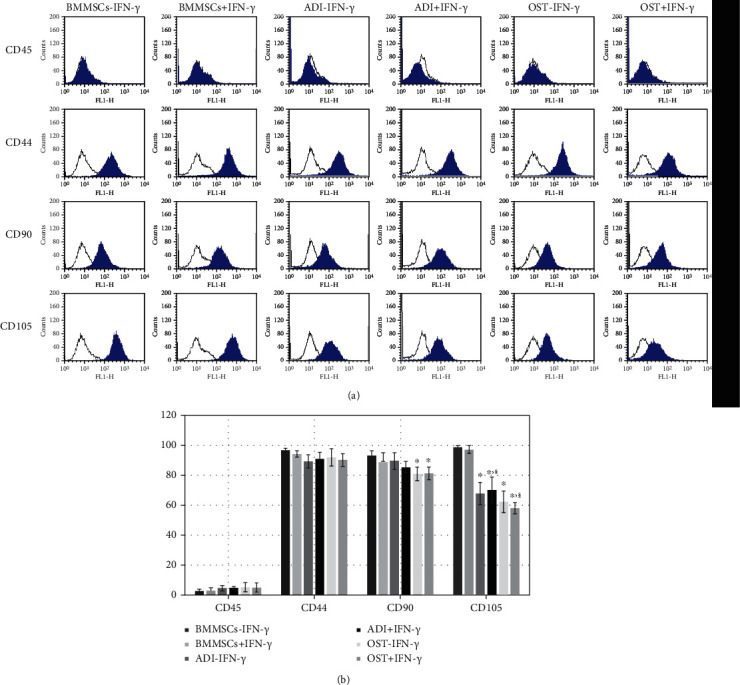
Assessment of MSC-specific surface molecule expressions of undifferentiated and differentiated MSCs with or without IFN-*γ* licensing. (a) Representative images of histograms for MSC-specific (CD44, CD90, and CD105) and hematopoietic (CD45) surface molecules detected by flow cytometry. (b) Statistical assessment of surface molecule expression in six groups (BMMSCs-IFN-*γ*, BMMSCs+IFN-*γ*, ADI-IFN-*γ*, ADI+IFN-*γ*, OST-IFN-*γ*, and OST+IFN-*γ*). Data represent the means ± SEM. *X* and *Y* axes show types of cells and positive cell population (%), respectively. ^∗^*p* < 0.01 indicated significant difference from BMMSCs-IFN-*γ*. ^§^*p* < 0.01 indicated significant difference between BMMSCs+IFN-*γ* and IFN-*γ*-licensed differentiated cells (BMMSCs+IFN-*γ* vs. ADI+IFN-*γ* or BMMSCs+IFN-*γ* vs. OST+IFN-*γ*). A significant difference between each differentiated MSCs and their IFN-*γ*-licensed cells was not found (*p* > 0.01; ADI-IFN-*γ* vs. ADI+IFN-*γ* or OST-IFN-*γ* vs. OST+IFN-*γ*.

The expressions of MSC-specific (CD44, CD90, and CD105) or hematopoietic (CD45) cell surface molecules in six groups were investigated (Figure 2(a)) and statistically assessed (Figure 2(b)). Undifferentiated MSCs (BMMSCs-IFN-*γ* and BMMSCs+IFN-*γ*) showed positive expression of MSC-specific cell surface molecules but negative expression of a hematopoietic marker, regardless of IFN-*γ* licensing; these results implied that the homogenous population of BMMSCs was isolated and used in the present study. In contrast, some of the MSC-specific cell surface molecules significantly (^∗^*p* < 0.01) reduced in differentiated MSCs during adipogenesis (CD105; BMMSCs-IFN-*γ* vs. ADI-IFN-*γ*) and osteogenesis (CD90 and CD105; BMMSCs-IFN-*γ* vs. OST-IFN-*γ*), which suggested the possibility that the features of MSCs were changed. Interestingly, IFN-*γ* licensing in differentiated MSCs was not able to increase the reduced expression of MSCs-specific cell surface molecules (ADI-IFN-*γ* vs. ADI+IFN-*γ* or OST-IFN-*γ* vs. OST+IFN-*γ*). And CD105 expression in IFN-*γ*-licensed groups was significantly (^§^*p* < 0.01) lower in differentiated MSCs than that in undifferentiated counterparts (BMMSCs+IFN-*γ* vs. ADI+IFN-*γ* or BMMSCs+IFN-*γ* vs. OST+IFN-*γ*).

### 3.3. Effect of IFN-*γ* Licensing in Expression of Immunosuppressive Molecules in Differentiated MSCs

**Figure 3:**
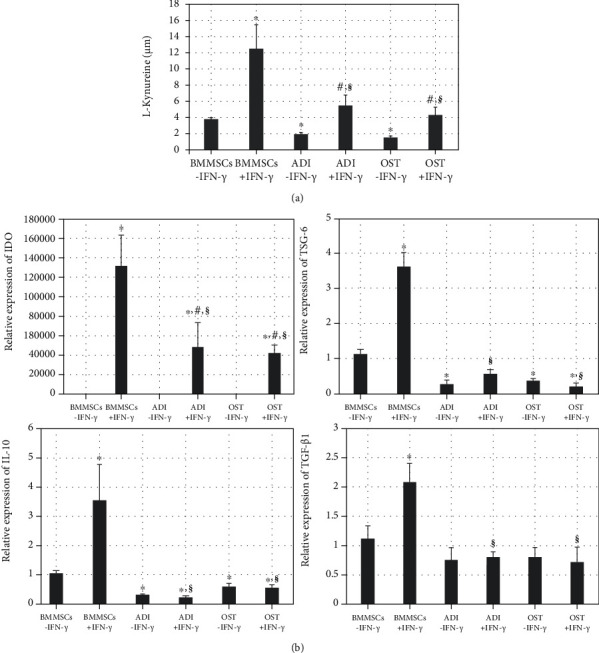
Effect of IFN-*γ* licensing in expression of immunosuppressive molecules in undifferentiated and differentiated MSCs with or without IFN-*γ* licensing. (a) IDO activity measurements using the level of L-kynurenine derived from six groups. Data represent the means ± SEM. *X* and *Y* axes presented types of cells and the concentration of L-kynurenine (*μ*M), respectively. (b) Expression of immunosuppressive molecules (IDO, TSG-6, IL-10, and TGF-*β*1) in the six groups by qPCR. Data represent the means ± SEM. *X* and *Y* axes represent types of cells and the relative expression of each gene, respectively. ^∗^*p* < 0.01 indicated significant difference from BMMSCs-IFN-*γ*. ^#^*p* < 0.01 indicated significant difference between each differentiated MSCs and their IFN-*γ*-licensed cells (ADI-IFN-*γ* vs. ADI+IFN-*γ* or OST-IFN-*γ* vs. OST+IFN-*γ*). ^§^*p* < 0.01 indicated significant difference between BMMSCs+IFN-*γ* and IFN-*γ*-licensed differentiated cells (BMMSCs+IFN-*γ* vs. ADI+IFN-*γ* or BMMSCs+IFN-*γ* vs. OST+IFN-*γ*). Abbreviations: IDO: indoleamine 2,3 deoxygenase; TSG-6: tumor necrosis factor-stimulated gene 6; IL-10: interleukin 10; TGF-*β*1: transforming growth factor beta 1; qPCR: quantitative polymerase chain reaction.

The relationship between expression of immunosuppressive molecules and IFN-*γ* licensing in differentiated MSCs was investigated by IDO activity measurements and gene expression analysis. IFN-*γ* licensing significantly (^∗^*p* < 0.01) induced marked elevation of IDO activity in undifferentiated MSCs (BMMSCs-IFN-*γ* vs. BMMSCs+IFN-*γ*). Although both types of differentiated MSCs exhibited significantly (^∗^*p* < 0.01) lower IDO activity than undifferentiated MSCs (BMMSCs-IFN-*γ* vs. ADI-IFN-*γ* or BMMSCs-IFN-*γ* vs. OST-IFN-*γ*), IFN-*γ* licensing to differentiated MSCs significantly (^#^*p* < 0.01) enhanced the IDO activity (ADI-IFN-*γ* vs. ADI+IFN-*γ* or OST-IFN-*γ* vs. OST+IFN-*γ*); however, enhancement of IDO activity in the differentiated MSCs by IFN-*γ* licensing did not produce the similar levels observed for IFN-*γ*-licensed undifferentiated MSCs (^§^*p* < 0.01; BMMSCs+IFN-*γ* vs. ADI+IFN-*γ* or BMMSCs+IFN-*γ* vs. OST+IFN-*γ*) (Figure 3(a)). In addition, when the mRNA levels of several immunosuppressive molecules (IDO, TSG-6, IL-10, and TGF-*β*1) were compared among the six groups, gene expression levels of IDO exhibited similar results to those from IDO activity, i.e., significant (^∗^*p* < 0.01) downregulation after differentiation of MSCs (BMMSCs-IFN-*γ* vs. ADI-IFN-*γ* or BMMSCs-IFN-*γ* vs. OST-IFN-*γ*) and significant (^#^*p* < 0.01) enhancement upon IFN-*γ* licensing in the differentiated state (ADI-IFN-*γ* vs. ADI+IFN-*γ* or OST-IFN-*γ* vs. OST+IFN-*γ*) (Figure 3(b)). The downregulated expressions of TSG-6 and IL-10 after differentiation were not affected by IFN-*γ* licensing in differentiated MSCs (*p* > 0.01; ADI-IFN-*γ* vs. ADI+IFN-*γ* or OST-IFN-*γ* vs. OST+IFN-*γ*), even though their expressions could be significantly (^∗^*p* < 0.01) increased after IFN-*γ* licensing in the undifferentiated state (BMMSCs-IFN-*γ* vs. BMMSCs+IFN-*γ*). IFN-*γ* licensing could significantly (^∗^*p* < 0.01) elevate TGF-*β*1 gene expression only in the undifferentiated MSCs (BMMSCs-IFN-*γ* vs. BMMSCs+IFN-*γ*). Overall, gene expressions of immunosuppressive molecules in IFN-*γ*-licensed differentiated MSCs were significantly (^§^*p* < 0.01) lower than IFN-*γ*-licensed undifferentiated state (BMMSCs+IFN-*γ* vs. ADI+IFN-*γ* or BMMSCs+IFN-*γ* vs. OST+IFN-*γ*).

### 3.4. Alteration of the Immunomodulatory Potential of Differentiated MSCs in a Xenogeneic Application

**Figure 4:**
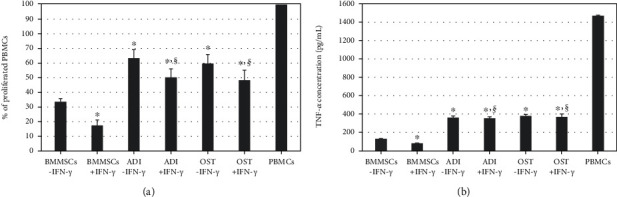
Alteration of the immunomodulatory potential of undifferentiated and differentiated MSCs with or without IFN-*γ* licensing in human PBMCs as a xenogeneic application by MLR and ELISA assay. (a) MLR assay with 1 × 10^5^ mitomycin C-treated MSCs for each of six groups and 1 × 10^6^ for PHA-stimulated human PBMCs, resulting in an MSC : PBMC ratio of 1 : 10. Data represent the means ± SEM. *X* and *Y* axes represent types of cells and the proliferated PBMCs (%), respectively; PHA-activated PBMCs seeded alone were counted as 100%. (b) An ELISA assay was used to detect the concentration of TNF-*α* in the supernatant post-MLR assay. Data represent the means ± SEM. *X* and *Y* axes present types of cells and the concentration of TNF-*α* (pg/mL), respectively. ^∗^*p* < 0.01 indicated significant difference from BMMSCs-IFN-*γ*. ^§^*p* < 0.01 indicated significant difference between BMMSCs+IFN-*γ* and IFN-*γ*-licensed differentiated cells (BMMSCs+IFN-*γ* vs. ADI+IFN-*γ* or BMMSCs+IFN-*γ* vs. OST+IFN-*γ*). A significant difference between each differentiated MSCs and their IFN-*γ*-licensed cells was not found (*p* > 0.01; ADI-IFN-*γ* vs. ADI+IFN-*γ* or OST-IFN-*γ* vs. OST+IFN-*γ*). Abbreviations: PBMCs: peripheral blood mononuclear cells; MLR: mixed lymphocyte reaction; ELISA: enzyme-linked immunosorbent assay; PHA: phytohemagglutinin; TNF-*α*: tumor necrosis factor *α*.

The immunomodulatory potential of undifferentiated and differentiated MSCs was assessed via an MLR assay with xenogeneic PBMCs and quantification of TNF-*α*, secreted by activated T cells, in the supernatant post-MLR assay. The MLR assay was conducted with coculturing of mitomycin C-treated MSCs as the effector and PHA-activated xenogeneic PBMCs as the responder at an MSC : PBMC ratio of 1 : 10. It was demonstrated that undifferentiated MSCs could suppress the proliferation of PBMCs, and their ability was significantly (^∗^*p* < 0.01) enhanced after IFN-*γ* licensing (BMMSCs-IFN-*γ* vs. BMMSCs+IFN-*γ*). In contrast, although both types of differentiated MSCs could inhibit the proliferation of PBMCs in part, the immunomodulatory property was significantly (^∗^*p* < 0.01) attenuated than that of undifferentiated MSCs in the xenogeneic application (BMMSCs-IFN-*γ* vs. ADI-IFN-*γ* or BMMSCs-IFN-*γ* vs. OST-IFN-*γ*). In addition, IDO activity between IFN-*γ*-licensed groups also exhibited significant (^§^*p* < 0.01) reductions after differentiation, compared with undifferentiated state (BMMSCs+IFN-*γ* vs. ADI+IFN-*γ* or BMMSCs+IFN-*γ* vs. OST+IFN-*γ*). The IFN-*γ* licensing mildly enhanced the reduced immunomodulatory property in differentiated MSCs (ADI-IFN-*γ* vs. ADI+IFN-*γ* or OST-IFN-*γ* vs. OST+IFN-*γ*), but it was not significant (*p* > 0.01) (Figure 4(a)). Similarly, the level of TNF-*α* in undifferentiated MSCs with IFN-*γ* licensing was significantly (^∗^*p* < 0.01) decreased in the supernatant post-MLR assay, compared with that derived from unlicensed undifferentiated MSCs (BMMSCs-IFN-*γ* vs. BMMSCs+IFN-*γ*). However, differentiated MSCs exhibited significantly (^∗^*p* < 0.01) increased TNF-*α* levels regardless of IFN-*γ* supplementation (BMMSCs-IFN-*γ* vs. ADI-IFN-*γ*, ADI+IFN-*γ*, OST-IFN-*γ*, and OST+IFN-*γ*). And in IFN-*γ*-licensed groups, the level of TNF-*α* was significantly (^§^*p* < 0.01) higher in differentiated state (BMMSCs+IFN-*γ* vs. ADI+IFN-*γ* or BMMSCs+IFN-*γ* vs. OST+IFN-*γ*) (Figure 4(b)). Overall, even though the expression and activity of an immunosuppressive molecule (IDO) in IFN-*γ*-licensed differentiated MSCs was enhanced (Figure 3), IFN-*γ* was incapable of elevating the reduced immunomodulation properties of differentiated MSCs in a xenogeneic application.

### 3.5. Changes in the Migratory Abilities of Differentiated MSCs in a Xenogeneic Application

**Figure 5:**
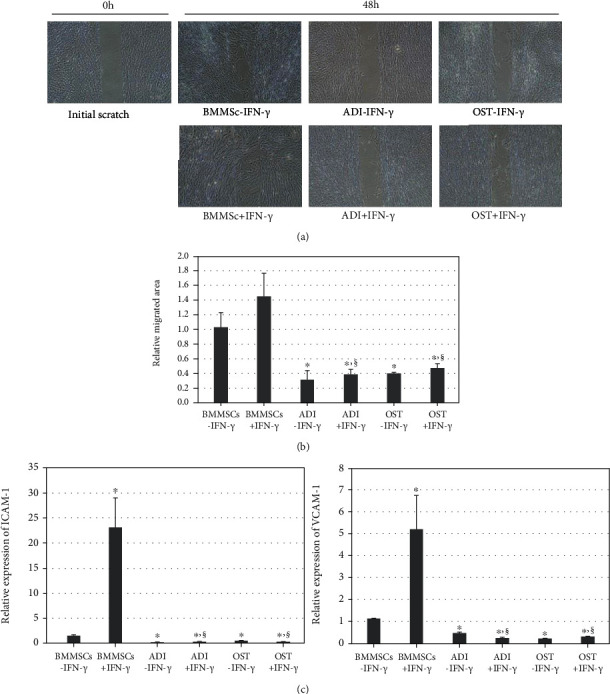
Change in the migratory ability of undifferentiated and differentiated MSCs with or without IFN-*γ* licensing, as detected with a scratch assay and gene expression analysis. (a) Representative images of the scratch assay for the six groups. After inducing an initial scratch at time point 0 h, MSCs were allowed to migrate to the scratched area for 48 h. Magnification: ×100. Scale bar: 100 *μ*m. (b) Statistical assessment of migratory ability of the six tested groups. The migrated areas postscratch were normalized to the value obtained from BMMSCs-IFN-*γ* at the 48 h time point. Data represent the means ± SEM. *X* and *Y* axes present types of cells and the relative migrated area, respectively. (c) Gene expression of migration-related genes (ICAM-1 and VCAM-1) in the six groups, as detected by qPCR. Data represent the means ± SEM. *X* and *Y* axes presented types of cells and the relative expression of each gene, respectively. ^∗^*p* < 0.01 indicated significant difference from BMMSCs-IFN-*γ*. ^§^*p* < 0.01 indicated significant difference between BMMSCs+IFN-*γ* and IFN-*γ*-licensed differentiated cells (BMMSCs+IFN-*γ* vs. ADI+IFN-*γ* or BMMSCs+IFN-*γ* vs. OST+IFN-*γ*). A significant difference between each differentiated MSCs and their IFN-*γ*-licensed cells was not found (*p* > 0.01; ADI-IFN-*γ* vs. ADI+IFN-*γ* or OST-IFN-*γ* vs. OST+IFN-*γ*). Abbreviations: ICAM-1: intercellular adhesion molecule 1; VCAM-1: vascular cell adhesion molecule 1.

**Figure 6:**
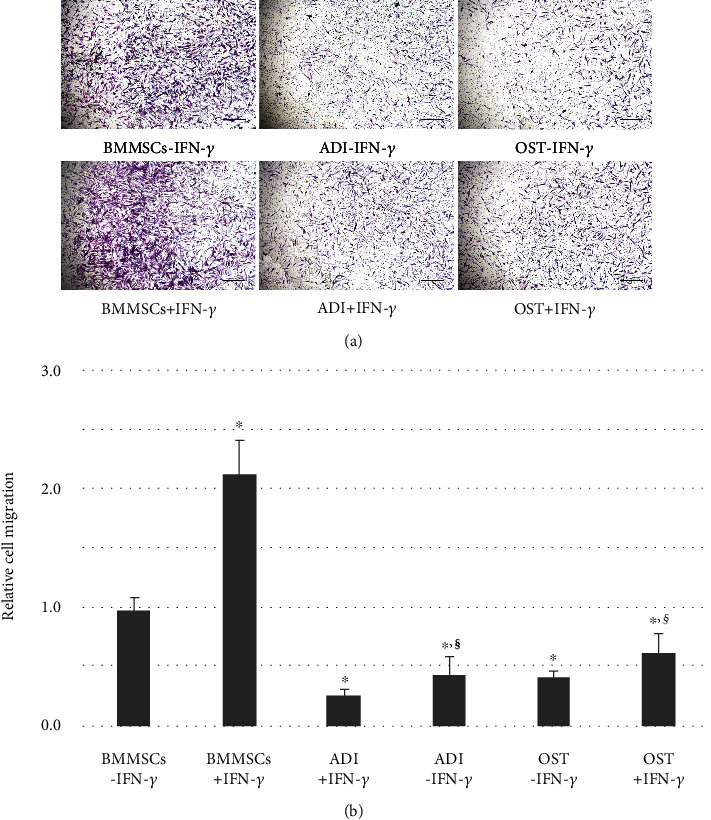
Changes in the migratory ability of undifferentiated and differentiated MSCs with or without IFN-*γ* licensing, as detected by a Transwell assay with human PBMCs as a xenogeneic application. (a) Representative images from the Transwell assay with MSCs from six tested groups in the upper chamber toward PHA-stimulated human PBMCs in the lower chamber. Crystal violet-stained cells indicate MSCs that migrated through membranes during 1 day. Magnification: ×100. Scale bar: 100 *μ*m. (b) Statistical assessment of the migratory ability of the six groups. The population of migrating MSCs was normalized to the value obtained from BMMSCs-IFN-*γ*. Data represent the means ± SEM. *X* and *Y* axes represent types of cells and the relative population of migrating cells, respectively. ^∗^*p* < 0.01 indicated significant difference from BMMSCs-IFN-*γ*. ^§^*p* < 0.01 indicated significant difference between BMMSCs+IFN-*γ* and IFN-*γ*-licensed differentiated cells (BMMSCs+IFN-*γ* vs. ADI+IFN-*γ* or BMMSCs+IFN-*γ* vs. OST+IFN-*γ*). A significant difference between each differentiated MSCs and their IFN-*γ*-licensed cells was not found (*p* > 0.01; ADI-IFN-*γ* vs. ADI+IFN-*γ* or OST-IFN-*γ* vs. OST+IFN-*γ*).

The migratory ability of undifferentiated and differentiated MSCs was assessed using a scratch assay, gene expression analysis, and a Transwell system with xenogeneic PBMCs. After inducing an initial scratch at the time point 0 h, undifferentiated MSCs exhibited strong migratory abilities, regardless of IFN-*γ* licensing (BMMSCs-IFN-*γ* and BMMSCs+IFN-*γ*) within 48 h. However, migration into the scratched area was significantly (^∗^*p* < 0.01) reduced in both types of differentiated MSCs (BMMSCs-IFN-*γ* vs. ADI-IFN-*γ* or BMMSCs-IFN-*γ* vs. OST-IFN-*γ*), and IFN-*γ* licensing did not enhance the reduced migratory ability in the differentiated MSCs (ADI-IFN-*γ* vs. ADI+IFN-*γ* or OST-IFN-*γ* vs. OST+IFN-*γ*) (Figures 5(a) and 5(b)). In the gene expression study, both types of differentiated MSCs expressed significantly (^∗^*p* < 0.01) lower mRNA levels of migration-related genes (VCAM-1 and ICAM-1) than undifferentiated MSCs, regardless of IFN-*γ* licensing (BMMSCs-IFN-*γ* vs. ADI-IFN-*γ*, ADI+IFN-*γ*, OST-IFN-*γ*, and OST+IFN-*γ*) (Figure 5(c)). In the Transwell system, the migratory ability of undifferentiated MSCs in the upper chamber toward the xenogeneic PBMCs in the lower chamber was identified and found to be significantly (^∗^*p* < 0.01) enhanced by IFN-*γ* licensing (BMMSCs-IFN-*γ* vs. BMMSCs+IFN-*γ*). In contrast, both types of differentiated MSCs presented significantly (^∗^*p* < 0.01) lower migratory abilities than those of undifferentiated MSCs (BMMSCs-IFN-*γ* vs. ADI-IFN-*γ* or BMMSCs-IFN-*γ* vs. OST-IFN-*γ*). Furthermore, although IFN-*γ* licensing in differentiated MSCs tended to increase the decreased migratory ability, this increase was not significant (*p* > 0.01; ADI-IFN-*γ* vs. ADI+IFN-*γ* or OST-IFN-*γ* vs. OST+IFN-*γ*) (Figures 6(a) and 6(b)). In IFN-*γ*-licensed groups of migratory ability assays, the values in differentiated MSCs was significantly (^§^*p* < 0.01) reduced in comparison with undifferentiated state (BMMSCs+IFN-*γ* vs. ADI+IFN-*γ* or BMMSCs+IFN-*γ* vs. OST+IFN-*γ*). Therefore, these results implied that IFN-*γ* licensing to the differentiated MSCs was incapable of enhancing the reduced migratory ability in a xenogeneic application.

## 4. Discussion

Since MSCs have the ability to differentiate toward several cell types, as well as immunomodulatory potential related to immune cells of both innate and adaptive immunity, MSC-based therapy has been subject to intensive investigation over recent decades in both preclinical and clinical studies [4, 9]. The immunomodulatory potential of MSCs can be achieved by secretion of anti-inflammatory factors including IDO, NO, PGE2, TSG-6, and TGF-*β*. The key immunomodulatory molecules that suppress immune responses by MSCs is species-dependent, i.e., IDO in humans, monkeys, and pigs, and inducible nitric oxide synthase (iNOS) in mice; the IDO degrades tryptophan to kynurenine in DCs and macrophages, which is followed by suppression of T cells and conversion of monocytes to M2-macrophages with secretion of immunomodulatory factors, such as IL-10 [8, 10]. Since pigs possess similar genetic and physiological characteristics as humans, porcine specimens including MSCs can provide valuable insights into therapeutic mechanisms and aid the development of promising therapeutic applications of MSCs to several human disorders as a xenogeneic regimen [7, 8]. However, whether differentiated MSCs retain their immunomodulatory properties in xenogeneic application models is still not clear, despite the knowledge that internal and external stimuli affect the potential of MSCs; MSCs applied to the inflammatory sites were directly exposed with proinflammatory cytokines [13, 18, 24]. Therefore, we aimed to reveal changes in the immunomodulatory and migratory properties of differentiated porcine MSCs and the effects of IFN-*γ* licensing on differentiated MSCs in an *in vitro* xenogeneic application model.

Differentiation of MSCs in the body plays a pivotal role in growth, tissue turnover, and the healing of damaged tissue [15]. Thus, *in vitro* differentiation induction to MSCs can be characterized as mineralization with upregulation of bone-related genes (OPN, ON, runt-related transcription factor 2, and osteocalcin (OC)) in osteogenesis and formation of intracellular lipid vacuoles with expression of fat tissue-related genes (PPAR-*γ*, FABP-4, and adipsin) during adipogenesis [15, 16, 25]. Likewise, porcine BMMSCs in the present study also presented the relevant cytochemical changes with upregulation of lineage-specific genes. Interestingly, licensing with IFN-*γ* affected the differentiation ability of MSCs at the mRNA level. In the same context, previous results have demonstrated that sustained exposure of IFN-*γ* to MSCs impaired adipogenic differentiation ability by inhibiting signal transducer and activator of transcription 3 activity in mice and downregulated expression of adipogenic gene, such as FABP-4 and adipsin in humans [25, 26]. Additionally, IFN-*γ* treatment in human BMMSCs exhibited downregulation of OPN, OC, and integrin-binding sialoprotein during osteogenesis [20, 25]. In addition to differentiation ability, expression of MSC-specific cell surface molecules, such as CD44, CD90, and CD105, is one of the major features of MSCs. However, these cell surface molecules are altered under differentiation induction. During chondrogenesis, human BMMSCs in 3-dimensional alginate culture exhibited highly decreased CD90 and CD105, and rat BMMSCs presented elevated expression of MHC-I, MHC-II, CD80, and CD86 [27, 28]. Differently expressed surface molecules from the membrane fractions between human BMMSCs and their differentiations were shown to have highly increased expression of CD10, CD92, and CD49e via mass spectrometry analysis, as well as decreased expression of CD105 in differentiated cells via flow cytometry [15]. In case of the effect of IFN-*γ* licensing to expression of MSC-specific surface molecules, both human BMMSCs and ATMSCs expressed similar levels of standard MSC-specific cell surface molecules, including CD44, CD73, CD90, and CD105, regardless of IFN-*γ* presence [6, 20]. Likewise, expression of MSC-specific cell molecules in the present study was investigated considering the aforementioned stimuli, differentiation induction and IFN-*γ* licensing. Results showed that some MSC-specific cell surface molecules (CD90 and CD105) were decreased after differentiation and that these alterations were not recovered by IFN-*γ* licensing.

The possibility that differentiation induction on MSCs is a potential obstacle during MSC applications in tissue replacement therapy has been proposed [11]. Therefore, several studies have investigated the compatibility of differentiation and immunomodulatory potential; however, the results are controversial. First, attenuation of immunomodulatory potential in differentiation-induced MSCs has been demonstrated under both conditions of allogeneic and xenogeneic applications. In cases of allogeneic experimental conditions, the differentiated rat BMMSCs toward chondrocytes lost their suppressive potential of T cell proliferation, including reduction of NO and PGE2 *in vitro*, as well as showing an increase in T cell infiltration after subcutaneous implantation into rat *in vivo* [27]. Furthermore, the loss of an inhibitory ability toward human PBMCs was found in differentiated human UCMSCs toward hepatocytes in the monolayer, and differentiated MSCs expressed lower PGE2 in comparison with that in undifferentiated cells [5]. In addition, while human BMMSCs suppressed the allogeneic neutrophil recruitment by TNF-*α*-treated endothelial cells through a collagen gel-based static adhesion assay, their adipogenesis was no longer able to inhibit neutrophil recruitment [18]. Moreover, although undifferentiated mouse BMMSCs were able to survive in the myocardium after being administered to an allogeneic myocardial infarction model, cardiac-differentiated MSCs induced immune reactions, such as severe infiltration of immune cells, caused by increased MHC molecules [29]. Similar results have also been found under xenogeneic experimental conditions. Differentiated rat BMMSCs toward chondrocytes were not able to inhibit the proliferation of human lymphocytes and induced cytotoxicity of lymphocytes via promoted human DCs relative to the effects of undifferentiated MSCs [21]. Porcine BMMSCs led to increased proliferation of human PBMCs after differentiation into hepatocyte-like cells [30]. In contrast, some studies have suggested that differentiated MSCs retain their immunomodulatory properties; however, even though undifferentiated and differentiated cells did not differ significantly in the general results for immunomodulatory potential, the enclosed data in these studies also presented weakened immunomodulatory properties after differentiation of MSCs. Although differentiated human BMMSCs and ATMSCs toward osteoblasts retained a suppressive ability toward allogenic PBMC proliferation, the ability was weakened in differentiated ATMSCs, though not significantly [12]. A previous study generally concluded that human BMMSCs during chondrogenesis could suppress the proliferation of T cells derived from rheumatoid arthritis (RA) patients; however, they also showed that the differentiated MSCs were unable to reduce the proliferation of T cells from the synovial fluid of RA patients in a ratio of 1 : 10 of an MSC : PBMC, which was the same proportion as that in the present study [31]. Furthermore, although differentiation induction in rat BMMSCs toward chondrocytes attenuated the inhibitory ability of proliferation of xenogeneic lymphocytes, such attenuation did not occur with adipogenesis and osteogenesis [21]. In agreement with the former findings, i.e., that differentiation induction altered the immunomodulatory properties of MSCs, the present study demonstrated that differentiation in porcine BMMSCs reduced IDO activity, decreased immunomodulation-related gene expression (IDO, TSG-6, and IL-10), and attenuated the inhibitory ability toward xenogeneic PBMC proliferation/activation. The discrepancies among studies may be attributable to several factors including the cell source of the MSCs, MLR systems with different effector/responder or their ratio, application strategies (allogeneic and xenogeneic), and the presence of proinflammatory cytokine licensing.

MSCs applied to the inflammatory sites are exposed with proinflammatory cytokines such as INF-*γ* and TNF-*α*, which is followed by licensing of MSCs [6–8]. It has been demonstrated that this INF-*γ* licensing in MSCs can enhance the immunomodulatory property in transplantation models. Human BMMSCs and bovine fetal BMMSCs were activated to express IDO via IFN-*γ* stimulation in a dose-dependent manner [10, 25]. Additionally, INF-*γ*-licensed human ATMSCs exhibited increased IDO transcription and decreased activated human T cell proliferation during coculture relative to these measures in unlicensed MSCs [6]. Furthermore, in a study conducted to identify the immunomodulatory potential of human MSCs *in vitro* and *in vivo* in response to IFN-*γ*, the expressions of CXCL9, CXCL10, CCL8, and IDO were highly upregulated after IFN-*γ* licensing to MSCs [32]. Likewise, the proliferation of allogeneic PBMCs was increasingly inhibited after coculturing with IFN-*γ*-licensed MSCs than with untreated MSCs. Furthermore, when transplantation of human PBMCs with MSCs was conducted in mouse as a xenogeneic GVHD model, improved immunomodulatory effects related to increased survival, decreased clinical symptoms, and less immune cell infiltration were found in IFN-*γ*-licensed MSCs-transplanted mice. In another xenotransplantation study, when a round clump of human BMMSCs was xenografted into a calvarial-defected mouse, unlicensed MSCs failed to regenerate the defective area with showing T cell infiltration in the grafted area, but INF-*γ*-licensed MSCs could present bone regeneration *in vivo* with higher IDO expression levels and suppression of T cells *in vitro* [20]. To the best of our knowledge, only three studies have assessed the effects of IFN-*γ* licensing on differentiated MSCs; notably, these experiments were only conducted in allogeneic application models. In human BMMSCs, INF-*γ*-exposed differentiated cells toward adipocytes and osteoblasts were enhanced to suppress proliferation of human PBMCs, as compared to differentiated cells not exposed to IFN-*γ* [33]. Human BMMSCs and ATMSCs retained the suppressive capacity of alloproliferation of PBMCs even after differentiation toward osteoblasts; this capacity in differentiated MSCs was enhanced after treatment of IFN-*γ* and TNF-*α* [17]. Likewise, IFN-*γ* licensing was thought to enhance immunomodulatory potential in differentiated MSCs in an allogeneic application model. However, in contrast to these two results, another study demonstrated that the immunosuppressive ability of differentiated rabbit BMMSCs to allogeneic lymphocytes was not enhanced after IFN-*γ* licensing; rather, they would deteriorate to a secretion of anti-inflammatory cytokine (TGF-*β*) [24]. The present study demonstrated the effect of differentiation induction in porcine BMMSCs with human IFN-*γ* licensing in a xenogeneic application with human PBMCs. Although differentiated MSCs could present increased IDO activity and gene expression after IFN-*γ* licensing, the level did not reach that of IFN-*γ*-licensed undifferentiated MSCs. In addition, differentiated MSCs caused less inhibition of the proliferation and activation of human PBMCs than did undifferentiated MSCs. Moreover, IFN-*γ* licensing could not enhance the reduced immunomodulatory potential of differentiated MSCs during a xenogeneic application. Especially, when MSCs are applied to inflammatory site, they are exposed with proinflammatory cytokines; the results from IFN-*γ*-licensed groups in the present study imply that the immunomodulatory potential of MSCs applied in the inflammatory site is attenuated if they are differentiated in the body during a xenogeneic application. To overcome these limitations, alternative ways for IFN-*γ* licensing to the cells are required. Recently, a promising method for IFN-*γ* licensing, multiple pulse stimulation of IFN-*γ* (MPS; 2 h at 10 ng/mL IFN-*γ* for 3 days), was presented to improve immune competence in the xenotransplantation of human pancreatic islet-like organoid (HILO) derived from human-induced pluripotent stem cells into diabetic mice; the MPS-HILO exhibited higher expression of programmed death-ligand 1, a major immune competence-related gene, was protected against IL-1*β*-induced *β*-cell dedifferentiation, and was functionally extendable in diabetic mice than single IFN-*γ*-licensed HILO (12 h at 10 ng/mL IFN-*γ*) [34].

Secretion of chemokine receptors and adhesion molecules in MSCs plays a pivotal role in immunomodulation [1, 9, 35]. MSCs are capable of migrating to injured tissues for the cell-to-cell contact with immune cells via expression of chemokine receptors/ligands such as CCR, CCL, CXCR, and CXCL [9]. In addition, adhesion molecules from MSCs including VCAM and ICAM are responsible for mediating cell-to-cell adhesion for inhibition of T cell activation and leukocyte recruitment to the inflammatory site; both genes were highly inducible with the presence of IFN-*γ* and inflammatory cytokines [6, 36]. During *in vivo* studies, migration of xenogeneic MSCs into the injured sites has been demonstrated as the presence of migrated MSCs after intravenous delivery in the infarct region of a rat myocardial infarction model of nonhuman primate BMMSCs [37], at the damaged site in a mouse allergic rhinitis model of human ATMSCs [38], and in a brain of rat cerebral ischemia model of human BMMSCs [39]. The migration ability of MSCs is commonly assessed *in vitro* using scratch assays and Transwell systems. Through these assays, it has been revealed that the migration ability of MSCs can be affected by several factors [6, 22, 23]. Senescent human BMMSCs in response to proinflammatory cytokines exhibited a reduced migratory ability [22]. The TNF-*α*-stimulated human ATMSCs showed an increased migration activity with expression of chemokine molecules in a Transwell system [23]. In contrast, INF-*γ* licensing in human ATMSCs did not induce change in migratory abilities during the scratch assay and VCAM-1 expression by flow cytometry, compared with unlicensed ATMSCs [6]. In addition, some cell adhesion-related genes (CD31 and E-cadherin) of MSCs were expressed at lower levels when they were cocultured with activated mouse splenocytes [36]. The present study showed that differentiation induction to porcine BMMSCs weakened the migratory ability under a scratch assay and Transwell system. In addition, although IFN-*γ* licensing took place to enhance the migratory ability of undifferentiated MSCs, it did not result in the positive effects in differentiated cells.

## 5. Conclusions

Taken together, the present results demonstrate that the immunomodulatory potential and migratory ability of porcine BMMSCs were comprehensively reduced after differentiation induction. In addition, IFN-*γ* licensing was incapable of enhancing the reduced potentials of differentiated MSCs in an *in vitro* xenogeneic application model. This study helps to improve understanding of utilizing MSCs therapy for immune-mediated disorders in the future.

## Figures and Tables

**Table 1:** Primer sequences for qRT-PCR.

Gene name (symbol)	Primer sequence (5′-3′)	Amplicon size (bp)	GenBank accession number
Fatty acid-binding protein 4 (FABP-4)	F: ggaaagtcaagagcaccataacc R: gctctctcataaattctggtagcc	160	NM_001002817
Peroxisome proliferator activated receptor *γ* (PPAR-*γ*)	F: ctcagacagattgtgacagagc R: tggaagaaggagaatgttgtcg	152	NM_214379
Osteonectin (ON)	F: tttggatggtttgtcgttctgc R: agagaaatgggctccttagtgg	163	NM_001031794
Osteopontin (OPN)	F: ggagcagtccaaagaatacacg R: tcttcttcgctcttagagtctgg	147	NM_214023
Indoleamine 2,3 dioxygenase (IDO)	F: gtgcagaagtggagaagttagc R: gagagtttgcagtaagggatgg	178	HM209418
Interleukin 10 (IL-10)	F: ctttagcaagctccaagagagg R: ccctagaatgcttcagttcttcc	118	NM_214041
Tumor necrosis factor-stimulated gene 6 (TSG-6)	F: tgaaattcctaagtgatgcttccg R: agttgtattctttccctgactgg	101	NM_001159607
Transforming growth factor beta 1 (TGF-*β*1)	F: ttaaaggtggagagaggactgg R: gaggaggaggaacaaatgaatgg	139	NM_214015
Intercellular adhesion molecule 1 (ICAM-1)	F: caacactttctccgacagatgc R: agcaaatccttgtttaccactcc	151	NM_213816
Vascular cell adhesion molecule 1 (VCAM-1)	F: agtgaactcttggaaagacatgg R: ctgatattgcaggcgatcttcc	196	NM_213891
TATA box-binding protein (TBP)	F: aacagttcagtagttatgagccaga R: agatgttctcaaacgcttcg	153	DQ845178

## Data Availability

The data used to support the findings of this study are available from the corresponding author upon request.
